# Effect of Culture Conditions on Viability of Mouse and Rat Embryos Developed *in Vitro*

**DOI:** 10.3390/genes2020332

**Published:** 2011-04-01

**Authors:** Elena Popova, Michael Bader, Alexander Krivokharchenko

**Affiliations:** Max-Delbrück Center for Molecular Medicine (MDC), Robert-Rössle-Str. 10, D-13125, Berlin-Buch, Germany; E-Mails: l.popova@mdc-berlin.de (E.P.); a.krivokharchenko@mdc-berlin.de (A.K.)

**Keywords:** preimplantation *in vitro* culture, culture system, oxygen tension, mouse and rat embryos

## Abstract

Currently *in vitro* culture of mouse preimplantation embryos has become a very important technique to investigate different mechanisms of early embryogenesis. However, there is a big difference in the preimplantation development between mammalian species. Despite close relatedness to mice, *in vitro* cultivation of rat preimplantation embryos is still delicate and needs further investigation and optimizations. In this study we have compared the *in vitro* developmental potential of mouse and rat embryos cultured at different culture conditions in parallel experiments. Interestingly, mouse zygotes developed *in vitro* until blastocyst stage even in inadequate medium without any phosphates and with low osmolarity which was formulated especially for cultivation of rat embryos. Rat parthenotes and zygotes developed in M16 medium formulated for mouse embryos only till 2-cell stage and further development is blocked completely at this stage. Moreover, developmental ability of rat embryos *in vitro* was significantly lower in comparison with mouse even in special rat mR1ECM medium. Mouse and rat embryos at 2-cell stage obtained *in vivo* developed until blastocyst stages significantly more efficiently compared to zygotes. Culture of mouse zygotes in glass capillaries resulted in a significantly higher rate of morula and blastocyst development compared with dishes. The Well-of-the-Well system resulted in a significant improvement when compared with dishes for the culture of rat zygotes only until morula stage. Reduced oxygen tension increased the developmental rate of rat but not mouse zygotes until blastocyst stage. This study demonstrates that development of early preimplantation embryos is altered by different culture conditions and show strong differences even between two related species such as mice and rats. Therefore, for understanding the fundamental mechanisms of early mammalian development it is very important to use embryos of various species.

## 1. Introduction

*In vitro* cultivation of mammalian ova is a very important tool to study early embryogenesis and environments in which the early embryo can undergo a number of divisions and form a blastocyst. The greatest success and considerable information concerning *in vitro* culture requirements for embryos of preimplantation stages have been reported for mouse embryos [1–3]. Later different *in vitro* culture systems have been developed for embryos for a variety of agricultural mammals including rabbit, sheep, cows and pigs [4–7]. Impressive results were also obtained with the *in vitro* manipulation of human embryos. The main finding was that there are great species-specific and stage dependent differences in sensitivity of embryos removed from the female reproductive tract to different *in vitro* culture compounds and conditions.

In spite of this, mouse preimplantation embryos are still the most popular model which is commonly used for investigation of mammalian early development *in vitro*. However, if we want to understand the fundamental mechanisms of preimplantation development we have to study other models in addition.

One of the most easily available and suitable mammalian species is rat. Notably, a lot of genetically characterized outbred and inbred rat strains have been generated and can be used for embryological studies. There are also other reasons for investigating early rat embryos *in vitro* since some very interesting peculiarities of rat preimplantation development *in vivo* and especially *in vitro* have been observed. As an intriguing example, a very special medium (mR1ECM) was developed for the culture of rat embryos from the 1-cell stage to the blastocysts [8]. This medium is significantly different from medium commonly used for *in vitro* culture of embryos of other mammalian species including human. Thus, mR1ECM unlike other media is absolutely phosphate-free. Moreover, even very small concentrations of inorganic phosphate ions completely blocked development of early stage rat embryos. Simultaneously, this medium has a very low osmotic pressure (240 *versus* 280–290 mosmol in conventional media) and increasing osmolarity inhibits development of early rat embryos.

Also the current situation in rat cloning is completely different compared to other mammalian species. All early attempts to generate cloned rats by transfer of nuclei from embryonic blastomeres have been unsuccessful [9,10]. Only recently we have reported the full-term development of rats after transfer of early cell cycle stage blastomere nuclei from 2-cell embryos into parthenogenetically pre-activated enucleated oocytes [11]. After a lot of unsuccessful attempts to obtain live offspring after somatic cell nuclear transfer in rat [12–16] one report about a cloned rat using somatic cells as karyoplasts has been published [17]. However, up to now, this study remained singular and more recent attempts were again unsuccessful [18,19].

Also, to our knowledge, there is the only one report available in the literature showing successful *in vitro* blastocyst development of cloned rat embryos [13]. In contrast, all other authors reported that somatic cell nuclear transfer (SCNT) rat embryos arrested *in vitro* at the 2-cell stage [15,16,18–20]. The reasons for this discrepancy are still not clear but are probably based on peculiarities of rat embryology and difficulties with the reproducibility of experimental conditions.

The aim of our work was to compare the effects of different culture media and culture systems on the development of early mouse and rat embryos *in vitro*.

## 2. Results and Discussion

### 2.1. *In Vitro* Development of Mouse Parthenogenetically Activated Oocytes, Zygotes and 2-Cell Stage Embryos in Different Culture Mediums

In our experiments we studied the developmental ability *in vitro* of mouse and rat embryos of different stages such as parthenogenetically activated oocytes, zygotes and 2-cell stages using in parallel two different culture media. We used culture medium (mR1ECM) which was established especially for *in vitro* culture of rat embryos from 1-cell stage to the blastocyst stage [8]. M16 medium was selected as the most commonly used conventional medium for culture of mouse embryos *in vitro* [21]. Parthenotes can be efficiently obtained *in vitro* with variety of mechanical, chemical, and electrical stimuli using oocytes of several species. Strontium is a very popular agent for parthenogenetic activation of mouse. In our previous experiments we also showed that rat parthenogenetically activated oocytes generated by strontium treatment successfully developed *in vitro* until blastocyst stage and were able to implant into the uterine wall after embryo transfer [22]. In these experiments we used established protocols for production of mouse and rat parthenotes and studied *in vitro* development until blastocyst stage in “rat” (mR1ECM) and “mouse” (M16) media using 4-well culture dishes.

As shown in Table 1, the rate of first cleavage of rat and mouse oocytes was not affected by the medium. However, the rate of development of activated mouse oocytes to the morula and blastocyst stages in mR1ECM (80.9 and 66.2%) was significantly lower compared with M16 medium (90.3 and 81.9%). Cells were counted in mouse blastocyst developed from activated oocytes in both groups. The number of cells in blastocysts developed in M16 medium was significantly higher (31.5 ± 1.9 (n = 11)) than in blastocysts cultured in mR1ECM (23.6 ± 2.1 (n = 9)) (P < 0.05). Rat parthenotes developed successfully until 2-cell stage after overnight culture in M16 medium (83.6%; 112/134) but none of them could develop further. However, 2-cell embryos transferred into mR1ECM developed until blastocyst stages (19.4%) but at lower rate than both groups of mouse parthenotes (66.2–81.9%) (P < 0.05). The number of cells in blastocysts from rat parthenogenetic blastocysts was significantly lower (14.8 ± 1.6 (n = 5)) compared to all other groups regardless of the medium used (P < 0.05).

We also assayed the ability of mouse and rat zygotes to develop *in vitro* using the same culture media and 4-well culture dishes which were previously described. As shown in Table 2, the rates of development to two-cell stage in mice did not depend on the medium (88.8–91.6%). However, the development to the morula and blastocyst stages and the mean number of cells per blastocyst were significantly lower for mouse zygotes cultured in mR1ECM compared to M16 medium (P < 0.05).

**Table 1.** Effect of culture medium on *in vitro* development of mouse and rat parthenogenetically activated oocytes.

**Medium and Species**	**No. of Activated Oocytes Cultured**	**No. of Parthenotes (%)**			**Cell Number in Blastocysts (mean ± SE)**
		**2-cells**	**Morula**	**Blastocyst**	
mR1ECM mouse	136	131/136(96.3)	110/136(80.9)*	90/136(66.2) *	23.6 ± 2.1(n = 9) +
M16-mR1ECM rat	134	112/134(83.6)	55/134(41.0)**	26/134(19.4)**	14.8 ± 1.6(n = 5) **
M16 mouse	72	72/72(100)	65/72(90.3)	59/72(81.9)	31.5 ± 1.9(n = 11)
M16 rat	134	112/134(83.6)	0/134(0)	0/134(0)	-

Values with different superscripts in the same column are significantly different.

* : Significantly different from M16 (P < 0.05);

+ : Significantly different from M16 (P < 0.05).

** : Significantly different from other groups (P < 0.001).

**Table 2.** Influence of culture medium on *in vitro* development of mouse and rat zygotes.

**Medium and Species**	**No. of Zygotes Cultured**	**No. of Embryos Developed into, (%)**			**Cell Number in Blastocysts (mean ± SE)**
		2-cells	Morula	Blastocyst	
mR1ECM mouse	152	135/152(88.8)	115/152(75.7)*	99/152(65.1)*	37.8 ± 1.7(n = 18)*
M16-mR1ECM rat	150	141/150(94.0)	90/150(60.0)**	49/150(32.7)**	19.5 ± 1.3(n = 22)**
M16 mouse	167	153/167(91.6)	140/167(83.8)	126/167(75.4)	45.0 ± 3.1(n = 21)
M16 rat	118	112/118(94.9)	0/118(0)	0/118(0)	-

Values with different superscripts in the same column are significantly different.

* : Significantly different from M16 group (P < 0.05);

** : Significantly different from other groups (P < 0.01).

Rat zygotes developed efficiently in M16 medium until 2-cell stage (94.9%; 112/118) without any differences compared to mouse zygotes (91.6%; 153/167). However, all embryos completely blocked at this stage like the parthenotes. When rat 2-cell stage embryos, after overnight culture in M16, were transferred into mR1ECM, they developed until morula and blastocyst stages (60 and 32.7%) but at significantly lower rate than the two mouse groups (75.7 and 65.1%) (P < 0.01). The number of cells in rat blastocysts developed *in vitro* was almost half (19.5 ± 1.3 (n = 22) that of the mouse embryos regardless of the culture medium used (37.8 ± 1.7 (n = 18) for mR1ECM or 45.0 ± 3.1 (n = 21) for M16).

We also recovered from mouse and rat females 2-cell stage embryos and cultivated them *in vitro* in mR1ECM and M16 media until hatched blastocysts. There was no difference in the capability of *in vivo* produced mouse 2-cell stage embryos to reach late blastocyst stage *in vitro* in both tested media (93.6–96.2%) (Table 3). However, we found dramatically lower rates of blastocyst hatching in mR1ECM medium −20.2 *vs.* 84.6 in M16. *In vitro* development of rat 2-cell stage embryos to the blastocyst stage was significantly less efficient than in mice. Only a few blastocysts (9.6%) started and none of them completed hatching from zona pellucida.

**Table 3.** *In vitro* development of mouse and rat 2-cell stage embryos in various mediums.

**Medium and Species**	**No. of 2-Cells Cultured**	**No. of Embryos Developed into, (%)**		
		**Blastocyst**	**Hatching Blastocyst**	**Hatched Blastocyst**
mR1ECM mouse	94	88/94(93.6)	18/94(20.2)	2/94(2.1)
mR1ECM rat	52	36/52(69.2)*	5/52(9.6)	0/52(0)
M16mouse	52	50/52(96.2)	44/52(84.6) **	33/52(63.5)**
M16 rat	44	0/44(0)	0	0

Values with different superscripts in the same column are significantly different.

* : Significantly different from other groups (P < 0.05);

** : Significantly different from all other groups (P < 0.001).

A lot of manipulations with rat embryos were limited for a long time by problems with the *in vitro* cultivation of preimplantation embryos in this species. The reasons for the developmental block of rat embryos cultured *in vitro* in M16 medium have been discussed many years ago. Kishi *et al.* reported the development of zygotes from Wistar rats to blastocysts *in vitro* in HECM-1 medium with rates of 10–20% depending on oxygen concentrations [24]. However, Ouhibi *et al.* could not obtain blastocysts from Wistar zygotes and only 5% of one-cell embryos from CD rats developed to blastocysts in HECM-1 medium [25]. In contrast, Matsumoto and Sugawara reported the development of 60% blastocysts from zygotes and more than 90% blastocysts from two-cell embryos using Wistar rats and HECM-1 medium [26]. The best rate was reported by Miyoshi *et al.* [26,27], who used one-cell rat embryos and achieved to get 60–90% blastocysts in a modified R1ECM medium. That is why this medium has become most popular and useful for successful cultivation of rat preimplantation embryos until blastocyst stage *in vitro*.

Interestingly, mouse parthenogenetic embryos, zygotes and 2-cell stage, could develop until blastocyst stage even in inadequate rat mR1ECM medium but at a lower rate compared with M16. *In vitro* culture of mouse 2-cell stage embryos in M16 resulted in a higher hatching rate probably because of the presence of BSA and other important components in this medium. Rat parthenotes and zygotes developed in M16 medium only until 2-cell stage and then development was completely blocked. Moreover, developmental ability of rat embryos *in vitro* was significantly lower in comparison with mouse even in the special rat medium, mR1ECM. So far *in vitro* culture of rat preimplantation embryos until blastocyst stage in established culture conditions remains still suboptimal and needs further optimizations.

### 2.2. *In Vitro* Development of Mouse and Rat Embryos in Different Culture Systems

There are a lot of factors affecting the efficiency of *in vitro* development of embryos in various mammalian species. One of the most important is embryo density (number of embryos per volume of medium) during cultivation, influencing the embryo interaction with soluble factors. The culture medium may dilute some important autocrine/paracrine factors effecting the viability and embryo developmental performance *in vitro* [28–30]. Various *in vitro* culture systems for preimplantation mammalian embryos have been established and described in the literature [3,31].

In our experiments, with the aim of improving *in vitro* development of rat embryos until blastocyst stage and to compare the developmental ability of mouse and rat embryos, zygotes and 2-cells of both species were cultured *in vitro* until blastocyst stage using three different culture systems.

Nunc 4-well dishes with 0.7 mL culture medium and 10 to 20 embryos per well is one of the most popular and conventional *in vitro* culture system for embryos of different mammalian species. We have previously also used this system successfully for the cultivation of mouse and rat embryos after different micromanipulations [11,22,32–34]. Glass capillaries with 10–50 μl of culture medium and 10 embryos per capillaries have been described for *in vitro* culture of mammalian embryos by Brinster many years ago [3]. This culture system was optimized and used to study the development of mouse embryos *in vitro* in a protein-free medium depending on the number of embryos in a small volume of medium [30]. The Well-of-the-Well or WOW culture system is another approach in which embryos are cultured in small indentations or microwells formed on the bottom of a plastic culture dish. This system was first developed for the individual culture of zona-free embryos [35,36]. Moreover, it was also shown to be efficient for culturing of zona-intact cattle embryos [35]. In our experiments we cultured mouse and rat zygotes and 2-cell stage embryos individually, one in each microwell.

Table 4 shows the *in vitro* development of rat embryos using three different culture systems. The cleavage rate of zygotes was the same for all culture systems tested. However, culture of zygotes in the WOW system resulted in a significantly improved development until morula stage (70.4%; 50/71) compared with zygotes cultured in Nunc dishes (53.0%; 44/83) (P < 0.05). However, we did not find any significant differences in blastocyst development for these groups of zygotes. Two-cell stage rat embryos developed *in vitro* without any differences between three studied culture systems.

**Table 4.** *In vitro* development of rat embryos using various culture systems.

**Culture System**	**Stage of Embryos**	**No. of Embryos Cultured**	**No. of Embryos Developed into, (%)**		
			**2-cells**	**Morula**	**Blastocyst**
Nunc dishes	Zygotes	83	79/83 (95.2)	44/83 (53.0)	24/83 (28.9)
	2-cells embryos	74	74	62/74 (83.8)	53/74 (71.6)
Glass capillaries	Zygotes	54	50/54 (92.6)	31/54 (57.4)	14/54 (25.9)
	2-cells embryos	48	48	39/48 (81.3)	30/48 (62.5)
WOW	Zygotes	71	65/71 (91.5)	50/71 (70.4)*	25/71 (35.2)
	2-cells embryos	52	52	45/52 (86.5)	32/52 (61.5)

Values with different superscripts in the same column are significantly different.

* : Significantly different from zygotes cultured in Nunc dishes (P < 0.05).

Table 5 presents the results for the *in vitro* development of mouse embryos in different culture systems. The rates of cleavage of mouse zygotes were the same in all groups regardless of the culture system. However, culture of zygotes in glass capillaries resulted in a significantly more efficient development until morula and blastocyst stage (92.5 and 85.0%) compared with Nunc dishes (79.9 and 74.5%) (P < 0.05). Two-cell mouse embryos developed equally until morula and blastocyst stage *in vitro*.

**Table 5.** *In vitro* development of mouse embryos using various culture systems.

**Culture System**	**Stage of Embryos**	**No. of Embryos Cultured**	**No. of Embryos Developed into, (%)**		
			**2-Cells**	**Morula**	**Blastocyst**
Nunc dishes	Zygotes	110	106/110 (96.4)	87/110 (79.9)	82/110 (74.5)
	2-cells embryos	43	43	42/43 (97.7)	39/43 (90.6)
Glass capillaries	Zygotes	80	80/80 (100)	74/80 (92.5)*	68/80 (85.0)*
	2-cells embryos	50	50	49/50 (98.0)	46/50 (92.0)
WOW	Zygotes	78	76/78 (97.4)	69/78 (88.5)	61/78 (78.2)
	2-cells embryos	40	40	40/40 (100)	40/40 (100)

Values with different superscripts in the same column are significantly different.

* : Significantly different from zygotes cultured in Nunc dishes (P < 0.05).

It was also reported that culture in glass capillaries is an efficient method of culturing of zona-free rabbit embryos. The authors compared three different culture systems and concluded that this method allows for the regular development of a high percentage of early rabbit embryo stages and for the simplification of some of the experimental procedures used in embryological research [37]. The WOW system resulted in significant improvement when compared to the drop-under-oil culture of *in vitro*-matured and parthenogenetically activated porcine oocytes, *in vivo* derived mouse zygotes and *in vitro*-fertilized human zygotes [38].

Taken together, the WOW system resulted in a significant improvement when comparing the Nunc dishes for culture of rat zygotes only until morula stage. However, the percentage of zygotes which developed until blastocysts was not improved. In mice we found a positive effect on culture until blastocyst stage in glass capillaries compared with Nunc dishes. Probably, rat embryos do not produce enough autocrine or paracrine factors *in vitro* unlike embryos of other mammalian species. Therefore, it would be very interesting to study the *in vivo* viability of rat embryos cultured *in vitro* at different culture conditions after transfer to foster mothers.

### 2.3. *In Vitro* Development of Mouse and Rat Embryos under 5% or 20% Oxygen

Several studies have shown that mammalian embryos develop better at reduced oxygen tension [39,40]. Higher concentration of oxygen was found to be toxic, probably due to the formation of oxygen radicals which induce detrimental effect not only on *in vitro* development but also on embryo metabolis [41] and gene expression [42]. However, to our knowledge, only one study has been performed about the possible effects of oxygen tension on rat embryos [43] and little is known about possible species-specific differences in sensitivity to oxygen tension of mouse and rat embryos.

In this study we have compared the *in vitro* development of mouse and rat embryos at atmospheric oxygen concentration of 20% and reduced oxygen concentration of 5% using 4-well culture dishes in parallel experiments (Table 6). In these experiments we used conventional *in vitro* culture conditions established for rat and mouse embryos. Thus, mouse embryos were cultured from zygote until blastocyst stage in M16 medium, whereas rat zygotes were cultured overnight in M16 and then transferred into mR1ECM medium in Nunc 4-well culture dishes. There were no differences in blastocyst development for mouse embryos cultured below 5% O_2_ or under atmospheric oxygen. However, the proportion of rat embryos developed to the blastocyst stage was significantly higher at reduced oxygen tension.

**Table 6.** Effects of oxygen concentration on the *in vitro* developmental ability in rat and mouse zygotes.

**Species**	**Oxygen Tension**	**No. of Embryos Cultured**	**No. of Embryos Developed into Blastocysts, (%)**
Mouse (M16)	20%	36	28/36(77.8)
	5%	35	27/35(77.1)
Rat (M16-mR1ECM)	20%	89	25/89(28.1)*
	5%	90	58/90(64.4)

Values with different superscripts in the same column are significantly different.

* : Significantly different from all other groups (P < 0.05).

These data are comparable with results published quite recently showing that reduction of oxygen tension enhanced development of rat 2-cell embryos to blastocysts in Wistar rats [43]. It would be very interesting to study embryos developed at different oxygen concentrations in more detail including the estimation of the number of cells in the inner cell mass and trophectoderm and of gene expression analysis. Further studies are warranted to estimate the *in vivo* developmental capacities of rat embryos cultured at reduced oxygen tension *in vitro*.

## 3. Experimental Section

### 3.1. Animals

Female C57BL/6N mice (23–25 days old) were obtained from Charles River (Sulzfeld, Germany). Female Sprague–Dawley-Hannover (SD-Hann) outbred rats (26-day-old) were obtained from a commercial animal breeder (Janvier, France). All mice and rats were kept at a temperature of 21 + 28 °C in a 12 h light/dark cycle (lights on 6.00 a.m.–6.00 p.m.) with a humidity of 65 + 5%. All experimental protocols were performed in accordance with the guidelines for the humane use of laboratory animals by the Max-Delbruck Center for Molecular Medicine and were approved by the local Ethics Committee.

### 3.2. Mouse and Rat Embryo Collection

Immature mouse and rat females were induced to superovulate by intraperitoneal injection of gonadotrophins: 5 IU for mice or 15 IU pregnant mare's serum gonadotrophin for rats (Intervet, Unterschleißheim, Germany) followed 45–50 h later by 5 IU for mice or 30 IU hCG (Intervet) for rats [34,44]. Superovulated females were sacrificed by cervical dislocation. Ovulated oocytes were collected 16–18 h after the hCG injection. To obtain embryos superovulated females were mated overnight with males of the same strain on the afternoon of Day 0. The criterion for mating was the presence of a vaginal plug on the following morning (Day 1 of pregnancy). Superovulated females were sacrificed at 12 p.m.–2 p.m. on Day 1 to collect zygotes. The oocytes and zygotes were recovered from the excised oviducts into M2 medium (Sigma) containing 0.1% (w/v) hyaluronidase (Sigma) to remove cumulus cells. Mouse and rat *in vivo* produced 2-cells were recovered from the excised oviducts 45–48 hrs after hCG. Then, the ova were washed in M2 medium and used for manipulations.

### 3.3. Parthenogenetic Activation of Oocytes

Parthenogenetic activation of mouse and rat oocytes by strontium treatment was performed as described previously [22,34]. The oocytes were incubated for 30 min in Ca^2+^ and Mg^2+^-free M16 medium containing 2 mM Sr^2+^ at 37 °C in a CO_2_ incubator. To obtain diploid parthenogenetic embryos, oocytes from all experimental groups were cultured 7–8 h in the presence of 5 mg/mL cytochalasin B (Sigma). The efficiency of pronuclear formation was analyzed 10–12 h after treatment. The oocytes were observed under an inverted microscope with Hoffmann optics. Those oocytes that formed visible pronuclei were recorded as activated.

### 3.4. Embryo Culture

For *in vitro* development rat and mouse embryos were cultured under 5% CO_2_ in air at 37 °C in mR1ECM medium [27] and M16 (Sigma) [34]. To compare the development until blastocyst stage, rat embryos but not mouse embryos were overnight cultured in M16 medium and then transferred into mR1ECM. Previously, the culture medium was equilibrated with the gas phase and temperature in a CO_2_ incubator for 2–3 h. To study the possible effect of the culture system on *in vitro* development the embryos were transferred into three different culture systems: 10–20 embryos into 700 μL of the medium in 4-well culture dishes (Nunc) [22]; 10 embryos into 25 μL of the medium in glass microcapillaries under paraffin oil [30]; single embryos in small wells with approximately 250-μm depth formed in 4-well dishes by melting the bottom with heated steel rods—Well of the Well (WOW) system [35].

Ova showing compaction and blastocoele cavity formation were classified as morulae and blastocysts, respectively. Blastocysts were classified as hatching when they were leaving zona pellucida and hatched when have completely left from zona pellucida. To study the possible effects of oxygen tension on *in vitro* development, mouse embryos were cultured in M16 medium and rat embryos were cultured overnight in M16 and then transferred into mR1ECM. Atmospheric oxygen concentration of 20% and reduced oxygen concentration of 5% was compared in parallel experiments.

### 3.5. Estimation of Embryo Cell Number

Cultivated embryos were assessed for cell number at the blastocyst stage using modified air-drying technique [45]. Briefly, embryos were exposed to a hypotonic solution consisting of 25% culture medium and 75% deionised water for 40–50 min. Cells were spread and fixed using a 1:3 mixture of glacial acetic acid and methanol. Preparations were stained with Giemsa [32].

### 3.6. Statistical Analysis

The z-test was used to determine the significance of proportions and was calculated using the online resource http://www.dimensionresearch.com/resources/calculators/ztest.html. For comparison among three groups or more, data were evaluated by one-way analysis of variance (ANOVA) followed by multiple comparisons. The two-tailed Student's t-test was used to test for differences of means. A value of p < 0.05 was chosen as an indication of statistical significance.

## 4. Conclusions

Our results demonstrated that early preimplantation mouse embryos are very resistant to culture conditions and can not be a representative model for the effects of these conditions on *in vitro* mammalian development in general. On the other hand, early preimplantation rat embryos have marked peculiarities in their *in vitro* development. It would be very interesting and important to study subsequent development *in vivo* of rat embryos cultured *in vitro* at different culture conditions.

We propose that the metabolism of rat embryos and their gene expression profile is different from embryos of mouse and probably also of other mammals. These differences may contribute to the extraordinary difficulties researchers have faced when trying to clone rats.

Therefore, to understand the fundamental mechanisms of early mammalian development and genome reprogramming, it is not enough to investigate only mouse embryos. It is very important to also use embryos of other species. Hence, available genetically defined pre-implantation rat embryos can be one of the best additional and suitable models for various embryological experiments.
